# Pre- and Post-Operative Quality of Life in Patients with Osteoradionecrosis of the Jaw

**DOI:** 10.3390/cancers16122256

**Published:** 2024-06-18

**Authors:** Sven Otto, Shreeja Shreeja, Sara Carina Kakoschke, Mohammed Michael Albittar, Andreas Widenhorn, Tamara Katharina Kakoschke

**Affiliations:** 1 Department of Oral and Maxillofacial Surgery and Facial Plastic Surgery, University Hospital, LMU Munich, Lindwurmstrasse 2a, 80337 Munich, Germany; sven.otto@med.uni-muenchen.de (S.O.);; 2 Deggendorf Institute of Technology, European Campus Rottal-Inn, Max-Breiherr-Strasse 32, 84347 Pfarrkirchen, Germany; andreas.widenhorn@th-deg.de; 3 Department of General, Visceral, and Transplant Surgery, University Hospital, LMU Munich, Marchioninistrasse 15, 81337 Munich, Germany; 4 Economics and Quantitative Methods Department, International School of Management, Karlstrasse 35, 80333 Munich, Germany

**Keywords:** osteoradionecrosis of the jaw (ORNJ), quality of life, head and neck cancer, health-related quality of life, oral health quality of life

## Abstract

**Simple Summary:**

This prospective study assesses and analyzes the pre- and postoperative quality of life (QoL) of patients with osteoradionecrosis of the jaw (ORNJ) after head and neck cancer therapy. It compares the QoL of subgroups concerning age, gender, ORNJ stages, type of surgical ORNJ therapy, hospital durations and chemotherapy in history. Clinical findings, demographic data and risk factors of ORNJ patients are also represented. This study addresses the lack of scientific literature and emphasizes the significance of the surgical therapeutic approach in decision-making for tailored and optimal therapy.

**Abstract:**

Osteoradionecrosis of the jaw (ORNJ) is a feared complication following radiation therapy performed for oncological treatment of head and neck cancers (HNC). To date, there is no clear evidence regarding the impact of surgical treatment of ORNJ on the quality of life (QoL) of affected patients. However, understanding the significance of the surgical treatment approach and its effects on QoL is an essential factor in the decision-making process for optimal, individualized therapy. In this prospective clinical study, QoL was assessed in relation to health related QoL (HRQoL) and oral health related QoL (OHQoL) before and after surgical treatment of ORNJ using standardized questionnaires (EORTC QLQ-C30, QLQ-HN35, OHIP-14). The overall QoL scores as well as individual domains of the collected scales regarding functional and symptom-related complaints were statistically analyzed. Subgroups concerning age, gender, different risk factors and type of ORNJ therapy were compared using Kruskal Wallis test. In addition, clinical and demographic patient data were collected and analyzed. QoL improvement correlated with the type of surgical ORNJ and the length of hospitalization. Better QoL scores were achieved post-operatively regarding different symptoms like pain, swallowing and mouth opening. Long-term effects of radiation therapy remained visibly restrictive to QoL and worsen over time.

## 1. Introduction

Head and neck cancer (HNC) is the seventh most prevalent cancer globally [1,2]. It includes various cancer entities affecting the larynx, hypopharynx, nasopharynx, oropharynx, oral cavity, nasal cavity, paranasal sinuses, and salivary glands. Approximately 90% of cases are squamous cell carcinomas [3]. In addition to surgery, radiotherapy (RT) is considered a standard treatment option, which can be performed with curative, adjuvant, or palliative intent [4].

Despite improved radiation techniques towards more targeted and lower-dose procedures osteoradionecrosis of the jaw (ORNJ) is still a feared and serious complication of radiotherapy of HNCs, when the jaw lies within or close to the radiation field [5,6,7,8]. It usually affects the mandible, but can also occur less frequently in the maxilla. The mandible might be more susceptible after radiation due to its denser bone structure and reduced blood supply [9]. In ORNJ, typically devitalized and devascularized bone remains exposed for more than three months without a tendency to heal and without evidence of a persistent or recurrent tumor [9].

The pathophysiological process of ORNJ contains radiation-induced hypoxia, hypovascularity, and hypocellularity of the affected bone and the surrounding soft tissues [10,11]. The affected tissues are less resistant, heal more difficultly, and are more prone to inflammation. Superinfections with bacteria from the oral and skin flora are common. Ulcers, fistulas, trismus, pain, and sensory disturbances are among the clinical symptoms. Complications such as extensive abscess formation or pathological fracture may occur [8]. Radiographic images do not always correlate with clinical signs [9].

The severity of ORNJ can vary widely, ranging from asymptomatic cases to those causing substantial discomfort, disfigurement, and functional impairment. The bone can be superficially or deeply affected. Lesions can be localized or extensive. In literature, various classifications, and staging systems of ORNJ can be found [9,11,12,13,14,15,16,17,18]. In clinical practice, ORNJ is often classified into three stages according to Notani et al. [19]. In Stage I, ORNJ is limited exclusively to the alveolar bone. In Stage II, ORNJ involves the alveolar bone and/or the mandible above the nerve canal. In Stage III, ORNJ extends below the level of the nerve canal and is associated with an extraoral fistula and/or a pathological fracture [19].

The patients who are already functionally compromised due to the consequences of HNC suffer even more from impairments in eating, speech, and potentially aesthetics when affected by ORNJ. This often leads to social consequences as well. Risk factors that can trigger the occurrence of ORNJ include age, gender, tumors of the oral cavity, tumors in close proximity to bony structures, type and duration of radiation, site of radiation, presence of infection, dental status, quality of dental treatments, smoking, and alcohol consumption [8,20]. Dental restorations prior to the initiation of radiation therapy and regular dental follow-ups during and after radiation treatment reduce the risk of ORNJ manifestation [7,21,22].

Patient-related, tumor-related, and treatment-related factors influence the development of ORNJ. However, accurately predicting, preventing, and treating it remains challenging. Advanced stages of ORNJ are difficult to treat with non-surgical approaches and often require extensive bone resections [23]. In many cases, after radical resection, reconstruction with microvascular re-anastomosed free flaps, such as from the fibula, is required [24]. However, the results of surgical procedures can be negatively affected by general consequences of radiation therapy, such as impaired wound healing and increased susceptibility to infections [25]. Furthermore, sequelae of radiation therapy such as tissue fibrosis, xerostomia, impairments of taste and smell, and functional issues like swallowing and speech disorders can also progress despite surgical treatment of ORNJ [23,24].

Although ORNJ has a significant impact on quality of life (QoL) of affected patients, there is a lack of focused research in this area. Various studies assess the QoL in HNC patients who generally have undergone radiation therapy, but without differentiating or focusing on those who developed ORNJ [26,27,28,29,30,31,32,33]. Some studies specifically assess the QoL of patients with ORNJ [34,35,36] with a few that take the therapy of ORNJ into account [37,38,39,40]. After surgical therapy of ORNJ improvement was i.e., observed in oral intake and speech, but persistent effects from (chemo)radiotherapy and post-surgery complications were associated with poorer outcomes, underscoring the need for careful management and expectation setting [37].

Understanding the value of a surgical therapeutic approach and its effects on patients’ QoL is an essential factor in decision-making for optimal and individualized therapy. However, there is no clear evidence so far, regarding the impact of surgical treatment of ORNJ on the QoL. Until now there is no study that captured the QoL of ORNJ patients before and after surgical therapy taking all ORNJ stages into account.

Therefore, the aim of this prospective study was to evaluate the impact of surgical therapy of ORNJ on the QoL of affected patients by providing a direct pre- and postoperative comparison. The QoL was assessed by standardized questionnaires before and after intervention on patients who underwent surgical ORNJ treatment from 2019 until 2022. For health related QoL (HRQoL) the EORTC QLQ-C30 [41] and QLQ-H&N35 [42] questionnaires were used; for oral health related QoL (OHQoL) the OHIP-14 questionnaire [43] was used. In addition, clinical and demographic data were collected and analyzed.

## 2. Materials and Methods

### 2.1. Methodology, Implementation, and Study Population

This monocentric, clinically prospective exploratory study was approved by the Ethics Committee of Ludwig-Maximilians-University (LMU) Munich (protocol code: 18-895; date of approval: 18 February 2019). The study population comprises patients diagnosed with ORNJ after oncological RT in the head and neck region, necessitating surgical treatment of ORNJ on an outpatient, inpatient, or day-patient basis. Inclusion criteria dictate eligibility for individuals with (1) history of head and neck cancer, (2) history of radiation to the head or neck, (3) ORNJ-diagnosis, (4) age above 18 years, (5) ability to respond and complete the questionnaire, and (6) ability to provide informed consent. Histopathologically, a tumor recurrence has been ruled out in all patients. According to the patient records available to us, all patients received dental screening, and if necessary, dental restoration before radiotherapy. The extent of dental restoration could not be detailed sufficiently in retrospect.

The study commenced after positive Ethics vote on February 2019, and concluded on January 2022. Participants received an introductory letter, refusal options/consent details, along with the questionnaires. Questionnaires were independently completed by patients up to 7 days before surgery, with assistance provided if needed. Three to five months after surgery, the participants were summoned for follow-up-examination and completion of the same questionnaires as part of the postoperative evaluation. The collection of data regarding QoL was assessed using the following instruments: Oral Health Impact Profile (OHIP-14) [43], EORTC QLQ-C30 Version 3.0 [41], and QLQ-H&N35 head and neck-specific questionnaire [42]. The acquisition of clinical and demographic data was done through the patient records.

### 2.2. Health-Related QoL-Questionnaires: EORTC QLQ-C30 and EORTC QLQ H&N35

The EORTC QoL Questionnaire (QLQ) is a comprehensive tool designed to evaluate the HRQoL in cancer patients who are part of global clinical trials. The EORTC QLQ-C30 version 3.0 (QLQ-C30(V3)) comprises a combination of multi-item scales and single-item assessments [41]. It encompasses five functional scales (physical, role, emotional, cognitive, and social), three symptom scales (fatigue, pain, and nausea/vomiting), a global health status/QoL scale, along with several individual items to gauge additional symptoms frequently mentioned by cancer patients (such as dyspnea, loss of appetite, insomnia, constipation, and diarrhea) and the perceived financial impact of the illness. A specialized head and neck module (EORTC QLQ-H&N35) is further available [42]. This 35-item questionnaire evaluates the symptoms and side effects of the treatment experienced by patients dealing with head and neck cancer and social function and body image/sexuality. It comprises 7 multi-item scales (pain, swallowing, senses, speech, social eating, social contact, and sexuality) along with 11 individual items (teeth, opening mouth, dry mouth, sticky saliva, coughing, felt ill, pain killers, nutritional supplements, feeding tube, weight loss, weight gain). Both EORTC QLQ-C30 and QLQ H&N35 are assessed using a verbal Likert response scale ranging from 1 to 4, with options including ‘Not at all’, ‘A little’, ‘Quite a bit’, and ‘Very much’ or in a Yes/No format. Exceptions being the two Global Health Status (GHS)/QoL items which employ a numeric response scale from 1 to 7, with endpoints labeled as ‘Very poor’ and ‘Excellent’. All responses are linearly transformed to obtain domain scores in the range of 0 to 100. A higher score on a functional scale signifies robust or healthy functioning, while a high score on the global health status/Quality of Life (GHS/QoL) scale signifies a superior QoL. Conversely, a high score on a symptom scale or individual item points to a greater level of symptomatology or issues. For all items and scales in H&N35, elevated scores point to a greater level of issues or challenges [41,44,45,46].

### 2.3. Oral Health Impact Profile 14 (OHIP-14)

The third section included inquiries regarding oral health-related QoL (OHRQoL), as assessed by the German version of the OHIP-14 questionnaire (OHIP-G14) [43]. The OHIP-14 is a clinically validated psychometric test that considers seven criteria of OHQoL encompassing functional limitations, physical pain, physical disability, psychological discomfort, psychological disability, social disability, and handicap. Here participants provide responses based on the frequency of these impacts using a 5-point Likert scale, ranging from ‘never’, ‘hardly ever’, ‘occasionally’, ‘fairly often’, to ‘very often/every day’. OHIP-14 scores range from 0 to 56, calculated by adding the ordinal values for the 14 items. Domain scores can range from 0 to 8. Higher OHIP-14 scores indicate poorer OHRQoL, while lower scores signify better OHRQoL [47,48,49].

### 2.4. Statistical Analysis

The results of the three questionnaires were analyzed statistically. The domain scores for EORTC QoL scales were calculated as detailed by the EORTC QoL manual [50]. Internal consistency of the domain scores was verified using Cronbach coefficient α. Intercorrelation across the EORTC scales was calculated using Spearman’s method to verify discriminant validity similar to Sherman et al. [42]. Spearman correlation coefficients were calculated between every pre and post-operative EORTC domain score to verify if there was a statistically significant change in the score due to the operation. This analysis was repeated for different demographic and clinical subgroups. The Shapiro-Wilk test was applied to the calculated EORTC score data to check the normality. A Nonparametric Wilcoxon rank sum test was applied to examine if the change between two dependent variables, pre and post-surgery domain scores (for each domain as well as overall domains) was statistically significant. Further, the primary EORTC QLQ-30 module includes a General Health Status/Quality of Life (GHS/QoL) scale. The overall GHS improvement and EORTC H&N35 domain change were analyzed over multiple demographic and clinical subgroups using the Wilcoxon rank sum test. The GHS scale improvement across subgroups was further evaluated using the Kruskall-Wallis test.

The overall OHIP-G14 score was calculated by summing all the item scores similar to Miksad et al. [51]. The mean, median, and standard deviation (SD) of the overall score and individual item scores were calculated. Shapiro-Wilk test was done to check the normality of these items. The pre and post-score for each item and overall score for the entire cohort as well as demographic and clinical subgroups were compared using the Wilcoxon rank sum test for paired data.

All significant tests were 2-tailed, and *p* < 0.05 was considered significant. Statistical processing and data analysis were conducted using SPSS Statistics 22 (IBM). Graphical representations were done with GraphPad Prism Version 9.5.1.

## 3. Results

### 3.1. Demographic and Clinical Findings

**Figure 1:**
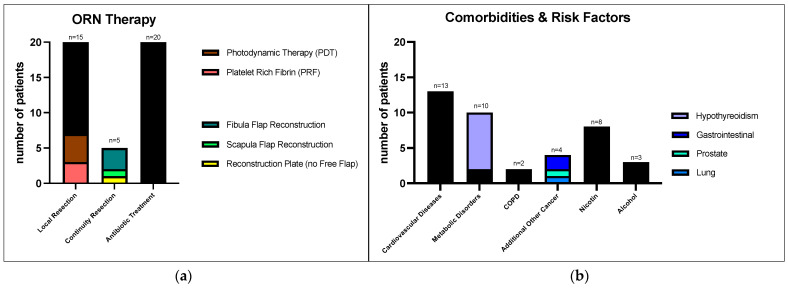
Therapy and Risk Factors of ORNJ: (**a**) Therapy conducted for the treatment of osteoradionecrosis: 15 patients underwent local mandibula resection without continuity resection, 5 underwent mandibula resection with continuity resection (out of these 3 received a mandibular reconstruction with a free fibula transplant, one with a free scapula transplant, and one received stabilization with a reconstruction plate without a free flap); (**b**) pre-existing conditions: number of patients with comorbidities and risk factors. Among the 10 metabolic disorders, 8 were hypothyreoidisms. Important subgroups are highlighted in color.

Between February 2019 and January 2022, twenty patients who underwent surgical treatment for ORNJ completed the questionnaires pre- and postoperatively. Among them were 15 men and 5 women, aged 58 to 88 years (mean age: 69.4 years). The demographic data and clinical findings are listed in Table 1. The type of ORNJ therapy is depicted in Figure 1a, and comorbidities as well as risk factors are shown in Figure 1b.

One patient had an angiosarcoma of the thyroid gland with cervical metastasis that was postoperatively irradiated, while all other patients had squamous cell carcinoma in the head and neck region, with 75% occurring in the oral cavity or oropharynx. Eleven (55%) patients received adjuvant radiation therapy after surgical tumor resection, eight (40%) patients received adjuvant radio-chemotherapy after surgical tumor resection, and one (5%) patient did not undergo tumor resection but received primary radio-chemotherapy. All twenty patients had a manifestation of ORNJ in the mandible, while one patient also had a simultaneous manifestation of ORNJ in the maxilla. Three (15%) patients were classified as Stage I, six (30%) patients were classified as Stage II, and eleven (55%) patients were classified as Stage III of ORNJ (Table 1) according to Notani [19].

Regarding the performed ORNJ therapy, five patients underwent partial mandibular resection with continuity resection, with four of them being reconstructed using microvascular re-anastomosed free flaps (three with fibula and one with scapula). One patient received a reconstruction plate without a transplant due to limited surgical capability. Fifteen patients underwent local mandibular resection without continuity resection, combined with modeling osteotomy. Among these patients, additional autologous PRF (platelet-rich fibrin) was used in three cases [52], and four received adjunct local photodynamic therapy using the Helbo^®^ low-level laser system, a diode laser with 670 nm wavelength and an output of 75 mW/cm^2^ combined with a photosensitizer dye containing methylene blue (Helbo^®^ Photodynamic Systems GmbH & Co KG, Senden, Germany) [53,54,55,56] (Figure 1a).

Five patients showed up with a pathological fracture of the mandible prior to surgical ORNJ treatment, and one patient developed a pathological fracture during the course after surgical necrosis removal. Three patients each had a preoperative extraoral fistula or an extensive purulent infection, respectively. Ten patients had a sensory disturbance of the inferior alveolar nerve, presenting as hypoesthesia or anesthesia prior to surgical ORNJ intervention. In seven patients, there was a persistent impairment of nerve function after surgical ORNJ treatment, with five of them undergoing a continuity resection of the mandible.

Four patients already had a PEG (percutaneous endoscopic gastrostomy) as feeding tube prior to ORNJ surgery. No patient received a permanent PEG placement after ORNJ surgery. Three more patients were postoperatively fed through a nasogastric tube just for a few days.

The average BMI was 21.05 kg/m^2^ preoperatively and 21.37 kg/m^2^ postoperatively. Nine patients gained weight, four lost weight, and seven maintained their weight (Table 1).

### 3.2. Differences in Pre- and Post-Operative QoL in ORNJ Patients

#### 3.2.1. Health-Related QoL Assessment with EORTC QLQ-30 and EORTC QLQ H&N35

**Figure 2:**
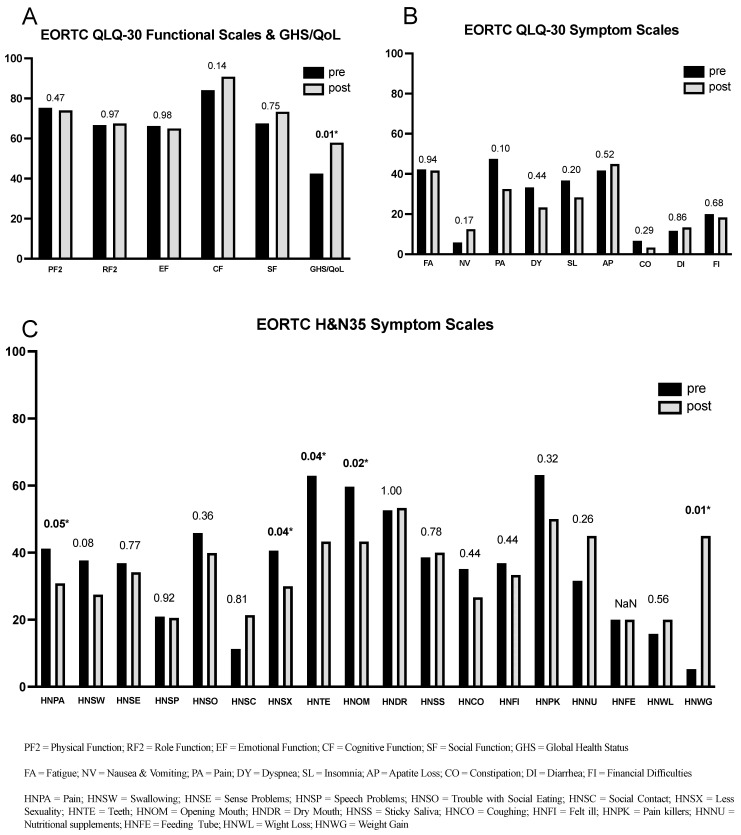
EORTC QLQ 30 and QLQ H&N 35 scores over functional and symptom scales pre-and post-surgery of ORNJ patients. *p*-values with Spearman’s correlation analysis, indicating statistical significance and suggesting a rejection of the hypothesis of identical distributions between pre- and post-surgery scores, are marked for scores with *p* < 0.05 with an asterisk (*). (**A**) pre- and postoperative scores from the EORTC QLQ 30 functional scales and GHS/QoL; (**B**) pre- and postoperative scores from the EORTC QLQ 30 symptom scales; (**C**) pre- and postoperative scores from the EORTC H&N35 symptom scales.

The scores for each EORTC domain are shown for pre- and post-surgery in Figure 2A,B. For assessing statistical significance of the post-surgery changes, the Wilcoxon test was utilized. A notable and significant enhancement in the GHS score was observed (*p* < 0.01), indicating an overall improvement in health and QoL following surgery.

In most functional as well as symptom scales, we saw that the post-operative scores were better than pre-operative scores, although the improvement was not always statistically significant, which might be attributed to small cohort size. So, for example, we saw increments of 5.66 for the cognitive functioning (CF) score (*p* = 0.14) and a reduction of 15 points in the pain symptom (PA) score (*p* = 0.10) post-surgery.

The domain scores for EORTC QoL H&N35 for both the pre- and post-operation are documented in Figure 2C. Most of the symptom scales here showed improvement post-surgery. There was a decrease in pain (HNPA, *p* = 0.05) and a reduction of 19.6 points in teeth-related problems (HNTE, *p* = 0.04). Additionally, there was a statistically better mouth opening ability reported by the patients (HNOM, *p* = 0.02) after surgery. The weight gain (HNWG) score matched the recorded information from the patient files (see Table 1), indicating a significant improvement (*p* = 0.01). Also an improvement in the HNSX-score (less sexuality) was reported by the patients (*p* = 0.04). Hardly no changes could be observed in items like dry mouth (HNDR) and sticky saliva (HNSS).

Some of the EORTC QoL scores are calculated based on multiple questions. In such cases, it is necessary to evaluate the consistency of the scores. For this purpose, the reliability coefficients (Cronbach α) were calculated for scores with multiple questions across both EORTC QLQ-30 and QLQ H&N35 scales. The result of this analysis is tabulated in Table 2. In the current analysis, most of the consistency values obtained were in the range from 0.72 to 0.99 indicating that the scores were reliable. As exceptions, only the post-operative social functioning scale had a reliability coefficient of less than 0.7 and was counted unreliable.

The correlations across different EORTC scales were calculated for the post-operative case to verify the validity of results. These are tabulated in Table 3. The correlations which had *p*-value less than 0.05 are marked in bold indicating statistically significant relations. As an example, we could see a high correlation (coefficient of 0.86) between FA (fatigue) and PA (pain) scales which could be expected. Similarly, there was a high negative correlation (−0.79) across PF2 (physical functioning) and PA (pain) scales indicating that the higher the pain experienced by the patient, the less was their physical functioning. Similar conclusions could be drawn by the relations in the QLQ-H&N35 scales. For example, the swallowing (HNSW) and trouble with social eating (HNSO) scales exhibited a high correlation (0.85) as expected. None of the QLQ-H&N35 scales exhibited a high degree of correlation with the trouble with social contact (HNSC) scale. This contrasts the correlation seen with the social functioning (SF) scale with the other scales in the core questionnaire. This indicates that the head and neck module offer independent information as compared to the core module.

Pain (PA) is negatively correlated with the GHS score pre- and postoperatively with a spearman coefficient of −0.42 (*p* = 0.03) and −0.57 (*p* = 0.004), respectively. The correlation is stronger postoperatively.

#### 3.2.2. Oral Health-Related Quality of Life Assessment with OHIP-14

The mean and SD of the OHIP-14 scores for the entire cohort are tabulated within Table 5 along with the summary scores of the different EORTC scales. Descriptive analysis for 14 questions based on OHIP dimensions were conducted for both pre- and post-operative patients in Table 4. None of the pre- and post-operative responses across questions were normally distributed as per Shapiro Wilk test. Hence, we also conducted Wilcoxon rank sum test to check if there are significant changes post-surgery. Although statistically not significant there were post-surgery improvements observed in pronouncing words; comfort while eating food; psychological comfort, regarding less self-consciousness, and anxiety. Nevertheless patients felt significantly more embarrassed after surgery (*p* = 0.03).

#### 3.2.3. Overall Improvement in ORNJ Patients after Surgery

There was an overall improvement in QoL and a positive correlation of pre- and post-operative results observed in the EORTC scales and OHIP G-14. Wilcoxon rank sum test showed great significance for the Global Health Status of the EORTC QLQ-30 questionnaire. (Table 5).

**Table 5:** Overall Improvement in postoperative QoL across EORTC Functional & Symptom Scales, Global Health Status Scale and OHIP G-14.

Domains	Pre Mean (SD)	Post Mean (SD)	Spearman Correlation	Wilcoxon *p*-Value
EORTC QLQ-30 Functional Scales	71.98 (±18.42)	74.13 (±17.00)	0.24	0.68
EORTC QLQ-30 Symptom Scales	27.28 (±17.54)	24.26 (±15.79)	0.40	0.43
EORTC QLQ-30 Global Health Status	42.50 (±14.02)	57.92 (±18.82)	0.08	0.01 *
OHIP G-14	21.42 (±12.84)	21.06 (±11.36)	0.45	0.80

Higher scores of the Global Health Status and the EORTC QLQ-30 Functional Scales indicate a better outcome, while lower values in the EORTC QLQ-30 Symptom Scale and OHIP G-14 stand for lower symptom burdens. *p*-values < 0.05 are marked with an asterisk (*).

### 3.3. Differences of QoL in Subgroups

#### 3.3.1. QoL Regarding the Type of ORNJ Therapy

To investigate whether treatment modalities and hospital duration have different effects on QoL of ORNJ patients, various subgroups were formed and examined.

While all patients showed improvement in terms of pain, mouth opening and teeth-related issues after surgery, interesting differences were observed concerning other oral health items and the Global Health Status, which related to the extent of the surgical intervention and hospital stay.

**Figure 3:**
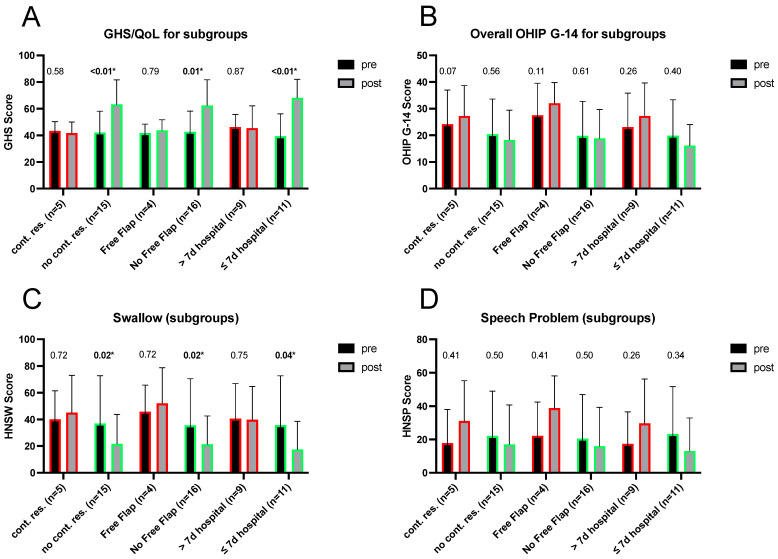
Pre- and post-operative comparison of subgroups regarding the overall GHS/QoL Score (**A**), the OHIP G-14 Score (**B**) and the EORTC-QLQ HN35 scores for swallowing (**C**) and speech problems (**D**). Significant *p*-values are marked with asterisks. Improvements are labelled in green and deteriorations in red.

The subgroups with no continuity resection and no free flap reconstruction, as well as the patients that stayed in hospital maximum 7 days for ORNJ-therapy showed statistically significant improvement of the overall GHS score post-surgery and swallowing problems (Figure 3A,C). Strikingly (although not statistically significant) the subgroups with continuity resections and a hospital duration longer than 7 days, tend to show a worsening in GHS score. The same trend can be observed for the subgroups with continuity resection, with free flap reconstruction and more than 7 days of hospital stay for the OHIP G14 score (Figure 3B) and speech problems (Figure 3D). For the aspects of dry mouth and sticky saliva, there were deteriorations in all subgroups.

#### 3.3.2. Demographic Differences in QoL

Next differences in age and gender were examined (Table 6). Patients above 70 years of age exhibited lower pre-operative GHS/QoL scores compared to patients below 70 years. However, this trend reversed in the postoperative GHS/QoL scores, with the older showing even higher values than the younger after surgery. Regarding symptoms, both age groups showed improvement post-operatively in the EORTC symptom scale, but in the OHIP as well as the EORTC H&N35 score, younger patients tended to report hardly no changes or even a slight increase in symptoms, while older patients showed a decrease. Also, the older age group (above 70 years) showed a functional improvement after the surgery on the EORTC functional scale.

Within gender subgroups, males exhibited superior scores in EORTC QLQ-30 functional scale and overall GHS/QoL status, coupled with lower scores in the EORTC QLQ-30 symptom scales before surgery. Postoperatively, males also show higher scores in the GHS/QoL and EORTC functional scale, and fewer symptoms in the EORTC symptom scale. However, when considering the difference between pre- and post-operative values, females show a greater improvement after surgery. Additionally, symptomatic complaints in the OHIP and H&N35 scores increased postoperatively in men, while they decrease in women. Consequently, male patients generally demonstrated higher QoL scores compared to females pre- and postoperatively. But interestingly, following surgery, female patients showed a greater enhancement in QoL compared to their pre-operative scores.

#### 3.3.3. Comparison of GHS/QoL Improvement between Subgroups

**Figure 4:**
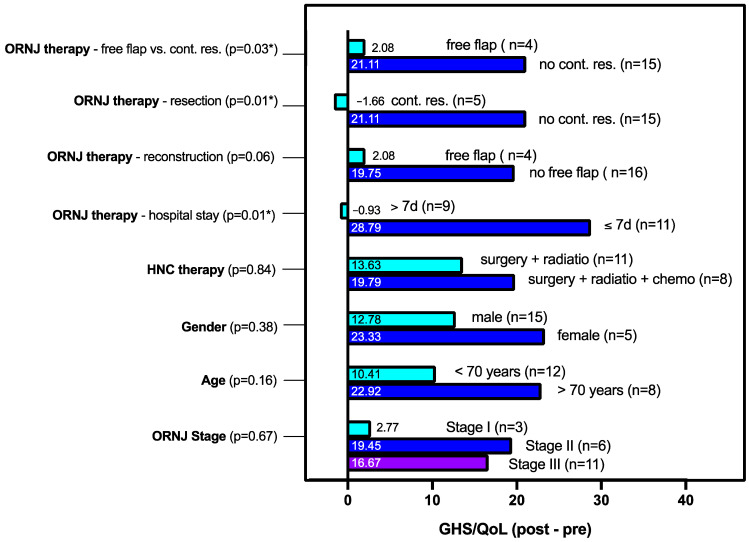
Comparison of GHS improvement of different subgroups using Kruskal Wallis test: Difference of post- and preoperative GHS/QoL scores are shown for various subgroups with corresponding *p*-value marked. The numbers of individuals in each group are added in brackets. *p*-values < 0.05 are marked with an asterisk (*).

To compare the extent of improvement in QoL, we conducted a detailed analysis of the change in GHS/QoL scores pre- and post-surgery, across different subgroups using the Kruskal-Wallis test (Figure 4). In addition to treatment modalities, we examined demographic aspects and the ORN stages.

Although there was an overall improvement in GHS/QoL scores for both genders, females exhibited a more substantial improvement compared to males, although this difference did not reach statistical significance (*p* = 0.38). This is probably due to the cohort size which was 15 males and 5 females.

Within the ORNJ therapy subgroups, intriguing distinctions emerged. Patients undergoing mandibular resection without continuity resection (15 individuals) demonstrated significantly better GHS/QoL improvement than those undergoing free flap reconstruction (4 patients) with a *p*-value of 0.03. When including the patient with the reconstruction plate without free flap reconstruction, there is even a slight deterioration in the GHS/QoL score of the group with continuity resection of the mandible compared to the group without continuity resection (*p* = 0.01).

When contrasting patients who underwent free flap reconstruction with all of those receiving no free flaps, the level of improvement closely parallels that observed in patients who underwent mandibular resection without continuity resection (*p* = 0.06).

Further exploration into the length of hospitalization, subgroup analysis revealed that patients hospitalized for less than 7 days experienced the highest GHS/QoL improvement, whereas patients who stayed 7 days or longer in hospital after ORNJ surgery showed decreased GHS/QoL scores postoperatively (*p*-value = 0.01).

Not significant (probably due to small cohort size), but still worth reporting are the results for age, ORNJ stage and previous HNC therapy. Age-wise categorization showed the subgroup above 70 years experiencing greater GHS/QoL improvement than the subgroup below 70 years of age (*p* = 0.16). Analyzing the different ORNJ stages the highest GHS/QoL improvement was observed in patients with ORNJ of stage II followed by ORNJ stage III (*p* = 0.67). Strikingly patients who had received chemotherapy in addition to surgery and radiotherapy (*n* = 8) as part of their previous HNC therapy performed better in terms of GHS/QoL improvement than those without chemotherapy (*p* = 0.84). This suggests that a more advanced HNC stage is not necessarily a hindrance to ORNJ therapy.

## 4. Discussion

Even a moderate tumor size extension in the head and neck region can impair speaking, swallowing, and oral food intake due to human anatomy. They can compromise the sense of taste and smell, lead to narrowing of the upper airways, cause pain, limit movement, and also cause unpleasant (oral) odor. As they increase in size and depending on their location, they can be also aesthetically disfiguring. It is not surprising that comprehensive QoL and oral health-related QoL are greatly affected by this type of cancer. The consequences of cancer treatment (surgery and reconstruction, radiotherapy, systemic therapies) also have their side effects and long-term consequences, further impacting the QoL. This includes aspects of oral health such as dental rehabilitation after loss of teeth or jaw parts, dry mouth and susceptibility to infections. Fears also come into play, such as the fear of tumor recurrence, financial worries due to inability to work or increasing costs related to therapy, and future concerns regarding oneself and one’s immediate environment, such as family. HNC patients also have an increased rate of depression or depressive disorders [57,58,59,60]. The need for tracheostomies or feeding tubes also increases social barriers.

Those patients who also develop ORNJ not only suffer from the symptoms and complications of ORNJ, but also from other long-term, often progressive effects of undergone cancer therapy (including also other side effects of radiation therapy), even if the ORNJ itself has been successfully treated. Therefore, it is hard to tell which aspect of reduced QoL are caused by ORNJ and which are linked to the underlying disease conditions.

In contrast to medication-related osteonecrosis of the jaw (MRONJ), ORNJ is significantly more challenging to treat in terms of outcomes and often requires more invasive treatment [61,62,63]. Extensive mandibular resections or repeated interventions may be necessary. When performing reconstructions with free flaps, one should also consider the longer duration of surgery, extended hospital stays, longer recovery time, and potential donor site morbidity.

Mucke et al. (2015) compared the QoL of patients undergoing different cancer treatments in the head and neck region. The study compared patients who only received surgery as part of their tumor treatment to those who also received radiation therapy. A third group included patients who developed ORNJ. When asked to reflect their QoL before and after cancer treatment, 56.3% of patients with ORNJ reported a strong deterioration compared to 31.3% of patients who underwent surgery only and 53.1% of patients who underwent surgery and radiotherapy but did not develop ORNJ [34]. Rogers et al. (2015) examined the QoL in 71 patients with different stages of ORNJ and compared them. They observed a significant deterioration in QoL in Stage III, according to Notani, and even recommended delaying surgical therapy with resection and reconstruction as long as the symptoms and pain are manageable and controllable [35].

The existing literature underscores the observed decline in QoL in ORNJ patients in clinical practice. Yet, there is limited information on how ORNJ-specific therapy influences QoL. Consideration arises regarding whether individuals already experiencing functional limitations should opt for comprehensive surgical and potentially highly invasive therapy which could add further temporary or permanent constraints. Alternatively, a symptomatic approach through best supportive care might be more appropriate, with surgery reserved for serious cases. In a study by Wang et al. (2009) however, the QoL was assessed in 15 patients who underwent resection with fibula reconstruction. The results showed that 70% of the participants reported an improvement in QoL after surgery. It was concluded that although long-term effects from radiation continued to affect QoL, reconstruction using the fibula flap allowed for better control of pain and local infections [39].

Chang et al. retrospectively analyzed the data of 35 patients who underwent surgical treatment for ORNJ between 1997 and 2007. Among them, 19 patients completed a QoL questionnaire after the treatment. The QoL after reconstruction with a free fibula transplant was compared to patients without reconstruction using a free flap (local debridement or debridement and fixation with a plate or reconstruction plate with a local flap). Despite high complication rates, most patients reported an improvement in their QoL after the operation [38]. In the study of Jacobson et al, 30 patients who had been treated for ORNJ in the mandible within a period of 6 years were interviewed via telephone postoperatively. The time interval between the interview and the surgical intervention varied in each case. Improvement was observed particularly in oral intake and speech, but persistent effects from chemoradiotherapy and post-surgery complications were associated with poorer outcomes, underscoring the need for careful management and expectation setting [37].

Another study conducted by Danielsson et al. focused on a cohort of patients who underwent free microvascular flap reconstruction of the mandible for a prospective comparison of pre- and post-operative QoL. Sixteen patients completed the EORTC QLQ-30 and QLQ-H&N35 questionnaires one month before the surgery and one year after the surgery. The results showed a tendency towards improved QoL, particularly due to pain reduction [40].

In our current study, we conducted a prospective assessment of QoL before and after the surgical intervention. However, we did not limit our analysis to patients who underwent free flap reconstructions but included all surgical treatments that were performed at our department. Over a period of two years, we collected pre- and post-operative questionnaires from 20 patients and monitored them during regular follow-ups after the therapy. A combination of three QoL questionnaires with different focuses was used. The EORTC QLQ-C30 was originally developed for cancer patients and includes general questions about physical complaints, emotional distress, social relationships and functional impairments. We supplemented it by the EORTC QLQ H&N35, which was specifically designed for patients with tumors of the head and neck region and includes questions that address symptoms and issues specific to this patient group, such as swallowing difficulties, speech problems and aesthetical impairments. The OHIP questionnaire generally targets oral health-related QoL. It can be used by individuals of any health status and can be employed to assess the outcomes of treatments and interventions. It is noteworthy that these questionnaires not only assess physical symptoms but also address biopsychosocial aspects, including well-being, anxiety, depression, perception of the illness experiences, feeling of shame, social support and questions about family situations.

We observed an overall improvement in the QoL with the most significant findings gained with the EORTC Global Health Status.

While pain, mouth opening and dental issues generally improved after ORNJ surgery, impairments with dry mouth and sticky saliva continued to increase. The latter are typical long-term effects of radiation that tend to worsen even after successful ORNJ therapy. There was a stronger negative correlation between pain and GHS score postoperatively, even though the overall pain score decreased after surgery. It is possible that patients had higher expectations for pain relief and QoL postoperatively. Therefore, any remaining pain might be perceived as more burdensome and have a greater negative impact on the GHS score. It could also indicate that after surgery patients might be more sensitive to pain, whether due to physical changes or increased awareness. Thus it is important to specifically manage any remaining pain to further improve the patients’ QoL.

Pain is a significant factor that influences QoL. Generally, however, we could not make any statements about the patients’ pain histories before ORNJ and pain perception is highly individual. Additionally, there are also other factors that can influence QoL, but are difficult to capture neither retrospectively nor within the questionnaires. These include for example the extent of dental restoration and rehabilitation prior or after radiotherapy, the precise tumor size and localization (proximity to nerves or other critical structures), social background and financial situation, job situation, activities and hobbies, fitness levels, mobility, sleep quality and much more. We documented and evaluated comorbidities, tumor entities, tumor stages and type of performed tumor therapy as well as nutritional status before and after ORNJ surgery. The kind and invasiveness of surgical therapy for ORNJ can vary greatly depending on the extent of necrosis and the overall condition of the patient and it is plausible that this can also influence QoL. Even though statistical analyses of subgroups have their limitations here, we want to address this heterogeneity and take the results and trends that emerged in this study into consideration for further evaluations.

Thus patients who underwent continuity resection or free flap reconstructions and those that stayed in hospital longer than 7 days reported no improvement in speech and swallowing compared to those with local resections and shorter hospital durations. These results are not surprising, given that these interventions are more extensive and mutilating. Additionally, since the post-operative QoL survey took place three to five months after the surgery, these patients were likely to be still in the logopedic rehabilitation phase. Nevertheless, this group also benefited from the relief of other symptoms, especially pain. However, when comparing the overall difference between postoperative and preoperative GHS/QoL-scores between the subgroups, it is noted that the gain in QoL after surgery is significantly greater for patients who underwent only a local resection without continuity resection of the mandible than for those with continuity resection or free flap reconstructions, despite similar pre-operative values (Figure 3B and Figure 4).

Another significant difference in comparing the subgroups of this study existed in that patients who spent less than 7 days in the hospital for the treatment of ORNJ experienced a clear increase in postoperative QoL, compared to those who spent 7 or more days in hospital. Typically, the latter are the more severely ill patients or those with greater surgical and nursing care needs, as well as those with postoperative complications.

Similar to the results of Rogers et al. (2015) [35] we saw that preoperative GHS/QoL score is higher the lower the ORNJ stage. However, in comparison to the improvement in QoL after ORNJ surgery, those of stage II and III performed better than those in stage I. Stage II benefited the most with the greatest gain in the GHS/QoL scores.

What also stood out was that age and a history of chemotherapy (which usually correlates with a higher stage of tumor disease) are not necessarily contraindications for surgical ORNJ therapy, as both the group of patients over 70 years old and the group of patients with previous chemotherapy in the context of HNC therapy showed even a higher increase in GHS/QoL after the ORNJ operation than those of lower age or those that had not received chemotherapy, respectively. We observed that in pre-operative cases functional scales and overall health-related QoL decline with advancing age, aligning with previous research findings [64,65]. However, post-operatively, there is a notable improvement in the overall health status scores among older individuals revealed in this study.

Previous studies on the QoL of ORNJ patients regarding the therapy showed small cohort sizes. Even though our prospectively designed study consists of a representative number of participants, it is also limited in some extent, as significance calculations were not always possible due to the number of 20 participants and even smaller numbers in the subgroups. But it provides interesting points for discussion and insights into the evaluation of QoL in ORNJ patients. There is a clear tendency towards improved QoL after surgery, also in severe cases of ORNJ. The authors point out that the decision for surgical ORNJ therapy and the type of surgical therapy must always be made in a case-by-case basis.

## 5. Conclusions

The choice and implementation of ORNJ surgery remains a significant challenge, but the fact that it is possible to improve the QoL of those affected gives hope despite high rates of complications and recurrences. Every patient him or herself is best able to assess what is important and essential for their QoL. This should always be taken into consideration during surgical management and medical care.

## Figures and Tables

**Table 1:** Demographic and clinical characteristics for the study cohort.

**Demographic data**	Age	Mean ± SD (years)	69.40 ± 9.18
		Min–max (years)	58–88
	Gender	Male	15 (75%)
		Female	5 (25%)
**HNC**	**Histopathology of HNC** Tumor Location	**Squamous Cell Carcinoma**	**19 (95%)**
		Oral Cavity and Oropharynx	15 (75%)
		Nasal Cavity	1 (5%)
		CUP/cervical metastasis	3 (15%)
		**Angiosarcoma**	**1 (5%)**
		cervical metastasis	1 (5%)
	**Treatment of HNC**	RT + Chemo	1 (5%)
		Surgery + RT	11 (55%)
		Surgery + RT + Chemo	8 (40%)
**ORNJ**	**Location of ORNJ**	Mandible	20 (100%)
		Mandible & Maxilla	1 (5%)
	**Site of ORNJ**	Right	10 (50%)
		Left	7 (35%)
		Bilateral or Midline Crossing	3 (15%)
	**ORNJ stage according to Notani et al. [19]**	Stage I	3 (15%)
		Stage II	6 (30%)
		Stage III	11 (55%)
	**Length of Hospitalization**	Mean ± SD (days)	9.65 ± 7.80
		Min–max (days)	4–31
**Nutritional status**	**BMI preop**	**Mean ± SD (kg/m^2^)**	**21.05 ± 2.73**
	BMI < 16	Critical underweight	1 (5%)
	BMI 16–18.5	Underweight	2 (10%)
	BMI 18.5–25	Normal weight	16 (80%)
	BMI 25–30	Overweight	1 (5%)
	**BMI postop**	**Mean ± SD (kg/m^2^)**	**21.37 ± 2.46**
	BMI < 16	Critical underweight	1 (5%)
	BMI 16–18.5	Underweight	1 (5%)
	BMI 18.5–25	Normal weight	17 (85%)
	BMI 25–30	Overweight	1 (5%)
	**Weight change**	gain	9 (45%)
		loss	4 (20%)
		equal	7 (35%)
	**Feeding tube**	PEG already present preoperatively	4 (20%)
		PEG present postoperatively	4 (20%)
		Temporary nasogastric tube postoperatively	3 (15%)
**Clinical Findings** **and** **Complications**	**Preoperative**	Infection	3 (15%)
		Extraoral Fistula	3 (15%)
		Pathological Fracture	5 (25%)
		Impairment of the inferior alveolar nerve	10 (50%)
	**Postoperative**	Infection/Wound Healing Disorder	2 (10%)
		Loss of Transplant	1 (5%)
		Pathological Fracture	1 (5%)
		Impairment of the inferior alveolar nerve	7 (35%)

RT = Radiotherapy, HNC = Head and Neck Cancer, ORNJ = Osteoradionecrosis, BMI = Body Mass Index, PEG = percutaneous endoscopic gastrostomy. Overarching aspects are in bold.

**Table 2:** Cronbach Reliability Coefficient Alpha for EORTC Scales ORNJ.

EORTC Domain	Post-Coefficient α
Physical Functioning (PF2)	0.83
Role Functioning (RF2)	0.72
Emotional Functioning (EF)	0.84
Cognitive Functioning (CF)	0.86
Social Functioning (SF)	**0.62**
Global Health Status/QoL	0.83
Fatigue (FA)	0.75
Nausea & Vomit (NV)	0.89
Pain (PA)	0.72
Pain (HNPA)	0.81
Swallowing (HNSW)	0.83
Senses Problem (HNSE)	0.98
Speech Problem (HNSP)	0.78
Trouble social eat (HNSO)	0.87
Trouble Social cont (HNSC)	0.91
Less Sexuality (HNSX)	0.98

Reliability coefficient value < 0.7 is marked in bold.

**Table 3:** Spearman Correlation between the EORTC scales for post- ORNJ.

Domain	PF2	RF2	EF	CF	SF	GHS	FA	NV	PA	HNPA	HNSW	HNSE	HNSP	HNSO	HNSC
PF2															
RF2	**0.61**														
EF	**0.56**	0.40													
CF	0.30	−0.07	0.38												
SF	**0.51**	**0.53**	**0.60**	0.19											
GHS	**0.59**	**0.52**	**0.54**	0.23	**0.46**										
FA	**−0.69**	**−0.64**	**−0.71**	−0.27	**−0.66**	**−0.73**									
NV	−0.43	−0.37	**−0.73**	−0.10	**−0.60**	−0.41	**0.50**								
PA	**−0.79**	**−0.59**	**−0.61**	−0.25	**−0.71**	**−0.57**	**0.86**	**0.55**							
HNPA	−0.18	−0.21	**−0.51**	−0.15	**−0.62**	−0.32	0.37	0.42	**0.47**						
HNSW	**−0.57**	−0.44	**−0.56**	−0.25	**−0.59**	**−0.67**	**0.57**	0.34	**0.58**	0.36					
HNSE	0.10	0.02	0.06	−0.02	−0.23	0.04	−0.19	−0.17	−0.02	**0.59**	0.18				
HNSP	−0.41	**−0.76**	−0.30	−0.08	**−0.57**	**−0.48**	**0.49**	0.23	**0.46**	0.34	**0.62**	0.27			
HNSO	**−0.48**	**−0.50**	**−0.64**	−0.13	**−0.66**	**−0.77**	**0.65**	0.34	**0.54**	**0.46**	**0.85**	0.17	**0.61**		
HNSC	−0.22	−0.32	−0.38	0.18	−0.38	−0.06	0.22	0.24	0.26	0.42	−0.03	0.22	0.29	0.27	
HNSX	**−0.64**	**−0.70**	**−0.63**	−0.21	**−0.62**	−0.37	**0.57**	**0.56**	**0.55**	**0**.46	0.39	0.15	**0.50**	0.40	**0.38**

Abbreviations in the column headlines refer to the corresponding scale name in the rows. Correlation values with *p*-value < 0.05 shown in bold. PF2 = Physical Function; SF = Social Function; PA = Pain; HNSP = Speech Problems; RF2 = Role Function; GHS = Global Health Status; HNPA = Pain; HNSO = Trouble with social eating; EF = Emotional Function; FA = Fatigue; HNSW = Swallowing; HNSC = Social contact; CF = Cognitive Function; NV = Nausea & Vomiting; HNSE = Senses problems; HNSX = Less sexuality.

**Table 4:** OHIP G-14 assessing the Oral Health Status before and after surgery across individual dimensions.

Dimensions	Questions	Pre		Post		Wilcoxon *p*-Value
		Mean	SD	Mean	SD	
Functional Limitation	Trouble pronouncing words	1.80	1.44	1.26	1.24	0.10
	Worsening of taste	1.90	1.45	1.89	1.52	0.95
Physical Pain	Feeling pain in mouth	2.50	1.61	2.11	1.37	0.41
	Discomfort when eating food	2.10	1.52	1.47	1.17	0.08
Physical disability	Unsatisfactory diet	1.55	1.32	1.63	1.38	0.69
	Interrupt meals	1.35	1.39	1.05	1.08	0.61
Psychological discomfort	Self-conscious	2.30	1.59	1.89	1.33	0.28
	Anxious	1.75	1.29	1.74	1.33	0.80
Psychological disability	Felt uncomfortable	1.32	1.20	1.53	1.43	0.30
	Felt embarrassed	0.70	0.80	1.26	1.10	0.03 *
Social disability	Irritable dealing with people	0.80	0.83	1.21	1.36	0.23
	Difficulty doing routine jobs	0.95	1.10	1.26	1.28	0.12
Handicap	Overall, less satisfying in life	1.90	1.45	1.58	1.26	0.29
	Unable to function	0.60	0.88	0.78	1.00	0.27

*p*-values < 0.05 are marked with an asterisk (*).

**Table 6:** Pre- and post-operative scores for age and gender subgroups.

**OHIP G-14 score**		**Pre**		**Post**		**Wilcoxon** ***p*-value**
		Mean Score	SD	Mean Score	SD	
Age	Below 70	21.00	±12.85	21.80	±10.81	0.15
	Above 70	22.00	±13.68	20.13	±12.71	0.53
Gender	Male	17.53	±11.51	20.23	±11.54	0.20
	Female	36.60	±3.37	23.20	±11.88	0.14
**EORTC QLQ-30 Global Health Status**		**Pre**		**Post**		**Wilcoxon** ***p*-value**
		Mean Score	SD	Mean Score	SD	
Age	Below 70	46.53	±11.49	56.94	±18.75	0.07
	Above 70	36.46	±16.02	59.38	±20.14	0.09
Gender	Male	46.11	±13.68	58.89	±20.28	0.07
	Female	31.67	±9.13	55.00	±15.14	0.04 *
**EORTC QLQ-30 Functional Scales**		**Pre**		**Post**		**Wilcoxon** ***p*-value**
		Mean Score	SD	Mean Score	SD	
Age	Below 70	75.80	±16.68	74.83	±16.17	0.88
	Above 70	66.25	±20.52	73.08	±19.27	0.40
Gender	Male	77.18	±17.39	75.84	±14.43	0.78
	Female	56.40	±12.18	69.00	±24.50	0.23
**EORTC QLQ 30 Symptom Scales**		**Pre**		**Post**		**Wilcoxon** ***p*-value**
		Mean Score	SD	Mean Score	SD	
Age	Below 70	27.88	±17.87	25.26	±14.36	0.53
	Above 70	26.39	±18.22	22.76	±18.66	0.48
Gender	Male	22.22	±16.60	22.18	±13.25	0.95
	Female	42.47	±10.62	30.49	±22.46	0.23
**EORTC QLQ HN35 Scales**		**Pre**		**Post**		**Wilcoxon** ***p*-value**
		Mean Score	SD	Mean Score	SD	
Age	Below 70	31.21	±18.83	36.94	±17.50	0.78
	Above 70	33.12	±24.08	29.25	±20.43	0.27
Gender	Male	25.11	±17.59	32.35	±20.44	0.17
	Female	54.72	±0.96	39.02	±11.59	0.11

Higher scores of the Global Health Status and the EORTC QLQ-30 Functional Scales indicate a better outcome, while lower values in the EORTC QLQ-30 Symptom Scale, EORTC H&N-35 and OHIP G-14 stand for lower symptom burdens. *p*-values < 0.05 are marked with an asterisk (*).

## Data Availability

The original contributions presented in the study are included in the article, further inquiries can be directed to the corresponding author.
